# Supplementary material for: Hybrid Models and Biological Model Reduction with PyDSTool
Source: PLoS Comput Biol. 2012 Aug 9;8(8):e1002628. doi: 10.1371/journal.pcbi.1002628 (PMC3415397; doi:10.1371/journal.pcbi.1002628)
Supplement: Text S4 — Complete source code for the PyDSTool package (version 0.88.120504). Includes API documentation and help files linking to web pages. This file is identical to the current public release on Sourceforge.net. (ZIP) [file pcbi.1002628.s004.zip › PyDSTool/html/PyDSTool.MProject.context-class.html]

xml version="1.0" encoding="ascii"?


PyDSTool.MProject.context


| Home | Trees | Indices | Help | PyDSTool |
| --- | --- | --- | --- | --- |

Package PyDSTool ::
Module MProject ::
Class context

[hide private]

[frames] | no frames]

# Class context

source code

```
object --+
         |
        context
```

---

A collection of related model interfaces that apply to a model.
interface\_pairs are a list of ModelInterface instance (test) and class
(ref) pairs, the latter to be instantiated on a model.

Set the debug\_mode attribute at any time, or as the optional argument
at initializiation, to ensure that any exceptions that arise from
interacting model interfaces and their features are fully passed back to
the caller of the context containing them.


|  |  |
| --- | --- |
| Instance Methods | [hide private] |

|  |  |
| --- | --- |
| \_\_init\_\_(self, interface\_pairs, debug\_mode=False)  x.\_\_init\_\_(...) initializes x; see x.\_\_class\_\_.\_\_doc\_\_ for signature | source code |

|  |  |
| --- | --- |
| reset\_weights(self, old\_weights=None)  Reset weights to unity, unless old\_weights array is given, in which case reset to that. | source code |

|  |  |
| --- | --- |
| set\_single\_feat\_weights(self, feat, weight=1)  Set weights for a single feature given as an (interface, feature) pair, setting all others to zero. | source code |

|  |  |
| --- | --- |
| set\_weights(self, weight\_dict)  Update weights with a dictionary keyed by test\_mi, whose values are either: (1) dicts of feature -> scalar weight. | source code |

|  |  |
| --- | --- |
| show\_res\_info(self, resvec)  Show detail of feature -> residual mapping for a given residual vector. | source code |

|  |  |
| --- | --- |
| \_map\_to\_features(self, x)  Utility to map 1D array x onto the model interface's features with non-zero weights, returning a dictionary. | source code |

|  |  |
| --- | --- |
| evaluate(self, model)  Evaluate whole context on a model instance, returning a single Boolean. | source code |

|  |  |
| --- | --- |
| residual(self, model, include\_raw=False)  Evaluate whole context on a model instance, returning an array of residual error between quantitative features in the model trajectory and their target values. | source code |

**Inherited from `object`**:
`__delattr__`,
`__getattribute__`,
`__hash__`,
`__new__`,
`__reduce__`,
`__reduce_ex__`,
`__repr__`,
`__setattr__`,
`__str__`


|  |  |
| --- | --- |
| Properties | [hide private] |

**Inherited from `object`**:
`__class__`


|  |  |
| --- | --- |
| Method Details | [hide private] |

|  |  |
| --- | --- |
| \_\_init\_\_(self, interface\_pairs, debug\_mode=False)  *(Constructor)* | source code |

x.\_\_init\_\_(...) initializes x; see x.\_\_class\_\_.\_\_doc\_\_ for
signature

Overrides: object.\_\_init\_\_: *(inherited documentation)*

|  |  |
| --- | --- |
| set\_weights(self, weight\_dict) | source code |

```
Update weights with a dictionary keyed by test_mi, whose values are
either:
 (1) dicts of feature -> scalar weight.
 (2) a scalar which will apply to all features of that model interface
Features and model interfaces must correspond to those declared for the
context.
```

|  |  |
| --- | --- |
| \_map\_to\_features(self, x) | source code |

Utility to map 1D array x onto the model interface's features with
non-zero weights, returning a dictionary.

x is assumed to have correct length.

|  |  |
| --- | --- |
| residual(self, model, include\_raw=False) | source code |

Evaluate whole context on a model instance, returning an array of
residual error between quantitative features in the model trajectory and
their target values.

Residual array will be weighted if one was set. Any weights set to
zero will cause those features to \*not appear\* in the residual.

Provide include\_raw=True argument to also return the raw, unweighted
residual. (Mainly for internal use.)

  


| Home | Trees | Indices | Help | PyDSTool |
| --- | --- | --- | --- | --- |

|  |  |
| --- | --- |
| Generated by Epydoc 3.0.1 on Fri May 4 15:24:06 2012 | http://epydoc.sourceforge.net |
